# Human parvovirus B19 infection in a renal transplant recipient: a case report

**DOI:** 10.1186/1756-0500-6-28

**Published:** 2013-01-23

**Authors:** Michelle Teodoro Alves, Sandra Simone Vilaça, Maria das Graças Carvalho, Ana Paula Fernandes, Luci Maria Sant’ Ana Dusse, Karina Braga Gomes

**Affiliations:** 1 Departamento de Biologia Geral, Instituto de Ciências Biológicas, Universidade Federal de Minas Gerais, Belo Horizonte, MG, Brazil; 2 Departamento de Análises Clínicas e Toxicológicas, Faculdade de Farmácia, Universidade Federal de Minas Gerais, Belo Horizonte, MG, Brazil; 3 Hospital Felício Rocho, Belo Horizonte, MG, Brazil

**Keywords:** Parvovirus B19, Anemia, Renal transplant, Antibodies

## Abstract

**Background:**

Parvovirus B19 presents tropism for human erythroid progenitor cells, causing chronic anemia in organ transplant recipients, due to their suppressed humoral and cellular responses. Diagnosis may be achieved through serological tests for detection of anti-B19 antibodies. However, renal transplant recipients are not routinely tested for parvovirus B19 infection, since there is scanty data or consensus on screening for B19 infection, as well as for treatment or preventive management of transplanted patients.

**Case presentation:**

Herein we report a kidney transplant recipient, who was unresponsive to treatment of severe anemia, and presented hypocellular hematopoietic marrow, megaloblastosis and hypoplasia of erythroid lineage with larger cells with clear nuclei chromatin and eosinophilic nuclear inclusions. This patient was seropositive for Epstein-Barr and Cytomegalovirus infections and negative for anti-parvovirus B19 IgM and IgG antibodies, although symptoms were suggestive of parvoviruses infection. A qualitative polymerase chain reaction testing for B19 in serum sample revealed positive results for B19 virus DNA.

**Conclusion:**

This case report suggests that the diagnostic process for parvovirus B19 in renal transplant recipients should include a polymerase chain reaction assay to detect B19-DNA, since specific serological tests may be unreliable given their impaired humoral responses. These results also indicate the importance of considering parvovirus B19 infection in the differential diagnosis of persistent anemia in transplanted patients.

## Background

The parvovirus (erythrovirus) B19 is a common human infection worldwide. The clinical manifestations of B19 infection depend on the host’s haematological status and immune responses [1]. In immunocompetent individuals, B19 causes the erythema infectiosum, also known as “fifth” disease. Classically, erythema infectiosum affects children who develop rash, fever and malaise, while in adults it may be associated with acute symmetrical polyarthropathy. B19 infection during pregnancy is associated with hydrops fetalis. In patients with chronic haemolytic anaemia, it correlates with transient aplastic crisis. In addition, it may also cause chronic anemia and pure red cell aplasia in immunocompromised patients [1,2].

The cellular receptor for B19 is a globoside (P antigen), present in erythroid precursor cells. The virus infects, replicates in, and then lyses erythroid progenitor cells [1]. This direct effect on erythroid cells manifests characteristically as pure red cell aplasia on bone marrow examination, revealing the presence of giant pronormoblasts, which can help the diagnostic process. B19 infection depends on mitotically active cells and susceptibility to infection increases in the erythroid precursors with differentiation [3]. Therefore, tissue distribution of the blood group P antigen helps to explain the extreme tropism of B19 for erythroid cells and the effects on hematopoiesis and bone marrow failure. Patients that do not present the P antigen in their erythrocytes are, therefore, resistant to infection by this pathogen [4].

Transmission of B19 infection occurs either by the respiratory route, vertically from mother to fetus, through transfusion, blood-derived products or transplantation. In immunocompetent patients, B19 infection is characterized by fever, chills and myalgia, which are followed by rash and joint symptoms [5].These later symptoms are associated to the appearance of specific antiviral antibodies. An effective immune response limits intense viremia in approximately 5 days. B19 specific immunoglobulin M (IgM) may persist for up to 6 months. Specific IgG is detectable about 2 weeks following infection and remains for years. Low reticulocytopenia occurs during viremia, but hemoglobin levels do not decline. In patients with chronic hemolytic disorders, transient aplastic crisis may occur during infection, since reticulocytopenia results in decreased hemoglobin levels. Nevertheless, the anemia is transitory resulting from development of specific antibodies against B19 antigens.

In immunocompromised patients, unable to mount humoral or cellular responses, B19 infection persists and may cause chronic anemia or erythroid bone marrow aplasia. Morphologically, bone marrow aspirates show giant proerythroblasts, large eosinophilic nuclear inclusion bodies, and cytoplasmic vacuolization [6].

Currently, diagnosis is based on detection of B19 IgG and IgM antibodies or B19 DNA in blood or tissue samples by polymerase chain reaction (PCR). A simple dot blot hybridization assay also detects infection; however the sensitivity of B19 detection is greatly improved by PCR. Immunohistochemistry is a specific alternative and may complement diagnosis in cases of placental or fetal infection [7-9].

## Case presentation

This case report describes to a Brazilian woman, 42 years-old, who presented with a renal failure and was submitted to haemodialysis for five years, before a kidney transplant, which occurred in 2007. After transplantation, the therapeutic regimen of immunosuppression included prednisone (5 mg daily), tacrolimus (5 mg daily) and azathioprine (50 mg daily). Dosage of serum tacrolimus was 5.8 ng/mL.

The donor’s B19 status for this recipient was unknown. In December 2010, the patient developed significant anemia, which was resistant to erythropoietin (1,119.0 mUI/mL) and, eventually, required blood transfusion. After transfusion, the patient’s hemoglobin was 6.8 g/dL and her hematocrit was 20.2%.

In April 2011, she presented cutaneous mucosa paleness, fatigue after minimal effort, arthropathy and malaise. She presented at the Hospital Felício Rocho, Belo Horizonte, MG, Brazil. Levels of hemoglobin and hematocrit were 3.6 g/dL and 10.3%, respectively. She received a transfusion of 600 mL of erythrocytes. Reticulocyte count was 7,200/mm^3^, leukocytes 4,100/mm^3^ and platelet 220,000/mm^3^. Dosage of serum creatinine was 2.3 mg/dL, iron (152 mcg/dL), transferrin saturation (89.9%), folate level (20.0 ng/mL), ferritin (938.6 ng/mL) and vitamin B12 (238.0 pg/mL), which did not suggest a nutritional or iron deficient anemia. Other laboratory investigations revealed she was seropositive for anti-Epstein-Barr (high IgG levels – 477.0 U/mL) and Cytomegalovirus (IgG positive) and negative for anti-hepatitis B, anti-hepatitis C and anti-HIV antibodies.

At this time point, a bone marrow aspirate revealed hypocellular for red and white cells and platelets. Besides, there were dysplasia and megaloblastosis in the erythrocytic series, which were attributed to azathioprine associated with tacrolimus toxicity.

A bone marrow biopsy was also obtained and showed severe hypoplasia of elements of the erythroid lineage, presence of larger cells with clear nuclei chromatin and eosinophilic nuclear inclusions, suggesting inclusions caused by B19. Nonetheless this evidence has indicated B19 infection, IgM and IgG assays were negative. However, as the symptoms and bone marrow biopsy were suggestive of B19 infection, a qualitative PCR testing for parvovirus B19 was performed, revealing the presence of this virus.

The woman received 5 doses of intravenous gammaglobulin, 400 mg/Kg body weight daily, which improved the symptoms. A new evaluation revealed an important increase in hemoglobin, from 3.6 to 12.6 g/dL.

## Discussion

In transplanted patients, parvovirus B19 infection is transmitted through the donor organ or contaminated blood products, during transfusion. Increased susceptibility due to immunosuppressive therapy is likely to favor establishment of infection [5,10]. B19 is not frequently regarded as a cause of anemia in immunosuppressed patients, although anemia without previous blood loss or reticulocytopenia should alert for potential B19 infection. Pure red cell aplasia and severe chronic anemia are also manifestations of B19 infection in organ transplant recipients and is directly related to the virus tropism for human erythroid precursor cells. Persistence of virus in bone marrow leads to prolonged suppression of erythropoiesis [10-12].

Although many cases of B19 infection in renal transplanted patients and various infection-related complications have been reported, only a few studies have been performed to evaluate the incidence of active B19 infection in anemic transplanted patients. Reported incidences of this infection vary from 23 to 31.1% of the cases. Given the prevalence of B19 infection in the general population (approximately 85% of older adults) and the increased susceptibility of transplanted patients to viral infection, it may be admitted that B19 infection is under-reported in this population [11,13-15].

This wide range of prevalence values reflects differences in definition of infection, in patient selection and in sensitivities of diagnostic methods. The diagnostic tests to detect anti-B19 IgM antibodies based on μ-capture sandwich enzyme immunoassay, display sensitivity of 89.1% and specificity of 99.4% [16]. The enzyme immunoassay for detection of IgG antibodies is reported to have a sensitivity of 98.6% and specificity 100% [17]. PCR and real-time PCR improve the sensitivity of detection of B19 infection, and many clinical laboratories use these molecular assays to complement the serologic diagnosis. Regarding the sensitivity of molecular methods, the Nested-PCR results in a thousand fold improved sensitivity when compared to conventional PCR. Since the qualitative detection of the DNA is not useful to confirm recent infection, real-Time PCR quantitative assays for viral DNA may be applied to differentiate acute from chronic infection [9,18,19]. Therefore, the interpretation of diagnostic test results is not always straightforward.

Moreover, in immunocompromised patients, false negative results may occur as due to depressed immune responses [15]. Egbuna *et al.*[11] described three transplanted patients with inadequate response to recombinant human erythropoietin treatment, under immunosuppressive therapy. One patient had negative serological tests for parvovirus B19, but a positive PCR, demonstrating the inability of the patient to mount a detectable effective anti-viral humoral response. Geetha *et al.*[12] performed a review of the literature for relevant articles of parvovirus B19 related anemia in solid organ transplant recipients, published between 1974 and 1999. Among the 14 cases reported, in which both serological and molecular tests were applied, all of them had positive PCR for B19. However, the IgM test was negative in two cases; the IgG test was negative in one and both IgM and IgG were negative in two cases. In the remaining nine cases, both serological and molecular tests were positive. Cavallo *et al.,*[13] described 48 renal transplant recipients with anemia. Eleven patients (23%) were positive for B19 DNA. However, ten were seropositive and one seronegative for the virus.

The case reported herein presented no evidence of hemolysis, blood loss or nutritional deficiency. The immunosuppression consisted of prednisone, azathioprine and tacrolimus. Persistent anemia and reticulocytopenia were observed and erythroid aplasia was established, even with erythropoietin use. Bone marrow biopsy revealed giant proerythroblasts and intranuclear inclusions, suggesting a chronic B19 infection, which may have been reactivated because of the immunosuppression. Furthermore, positive serological tests, i.e. anti-Epstein-Barr and anti-Cytomegalovirus, which are commonly seen in B19 disease, were also positive in this woman.

Organ transplant recipients are in risk of symptomatic B19 virus infections. However, the diagnosis may be complicated by low titer viremia and the absence of detectable humoral and/or cellular immune response, due to immunosuppressive therapy [13,15]. In the present case, despite the evidence of parvovirus B19 infection, the specific IgM and IgG were negative, requiring a qualitative B19 PCR in serum, which is more sensitive, to confirm the diagnostic hypothesis. These serological findings suggest that B19 infection has been acquired from the donor of the transplanted kidney and that immunosuppression did not allow the development of the patient’s antibody response. Nevertheless, the hypothesis of reactivation of B19 infection cannot be ruled out, since the donor or the patient’s infection status, previously to the transplant, is unknown.

Transplanted patients are immunossupressed to avoid host to graft reactions. However, the optimal treatment for B19 infection requires reduction of intravenous immunosuppressive drugs and administration of gammaglobulin, since immunosuppression may alter host defense mechanisms, leading to reactivation of the B19 virus or impaired immune responses, when infection is transmitted through the organ grafting. Although the B19-associated anemia can improve spontaneously, intravenous gammaglobulin is usually necessary in the majority of patients. However, it is still unknown whether the virus is completely eliminated after this treatment [12,20].

## Conclusion

Parvovirus B19 infection should be considered for the differential diagnosis of persistent anemia non responsive to erythropoietin, aplastic crisis and other opportunist infections in transplanted patients. The true incidence of this infection may be underestimated, because B19 serology may not be routinely searched in transplanted patients. Since serological tests may fail to detect B19 infection in immunosuppressed patients, addition of a polymerase chain reaction assay to detect B19 DNA should be considered to improve sensitivity and to guide adequate treatment.

## Consent

Written informed consent was obtained from the patient for the publication of this case report and any accompanying images. A copy of the written consent is available for review by the Editor-in-Chief of this journal.

## Competing interests

The authors declare that they have no competing interests.

## Authors’ contributions

MTA reviewed the patient’s medical record, analyzed data from the literature and wrote the article. SSV followed up the patient during the hospitalization and reviewed the manuscript. MGC, APF and LMSD analyzed data from the literature and reviewed the manuscript. KBG was the major contributor in writing the manuscript. All authors read and approved the final manuscript.
